# Novel multi jet fusion 3D‐printed patient immobilization for radiation therapy

**DOI:** 10.1002/acm2.13773

**Published:** 2022-09-02

**Authors:** James L. Robar, Barret Kammerzell, Kevin Hulick, Pierre Kaiser, Calvin Young, Vanessa Verzwyvelt, Xin Cheng, Matthew Shepherd, Radojka Orbovic, Sara Fedullo, Christopher Majcher, Stephen DiMarco, James Stasiak

**Affiliations:** ^1^ Department of Radiation Oncology Dalhousie University Halifax Nova Scotia Canada; ^2^ Nova Scotia Health Halifax Nova Scotia Canada; ^3^ HP Labs Corvallis Oregon USA; ^4^ HP Vancouver Washington USA; ^5^ HP Corvallis Oregon USA; ^6^ Adaptiiv Medical Technologies Halifax Nova Scotia Canada

**Keywords:** 3D printing, dosimetry, immobilization, multi jet fusion, radiation therapy

## Abstract

**Purpose:**

Thermoplastic immobilizers are used routinely in radiation therapy to achieve positioning accuracy. These devices are variable in quality as they are dependent on the skill of the human fabricator. We examine the potential multi jet fusion (MJF) 3D printing for the production immobilizers with a focus on the surface dosimetry of several MJF‐printed PA12‐based material candidates. Materials are compared with the goal of minimizing surface dose with comparison to standard thermoplastic. We introduce a novel metamaterial design for the shell of the immobilizer, with the aims of mechanical robustness and low‐dose buildup. We demonstrate first examples of adult and pediatric cranial and head‐and‐neck immobilizers.

**Methods:**

Three different PA12 materials were examined and compared to fused deposition modeling–printed polylactic acid (PLA), PLA with density lowered by adding hollow glass microspheres, and to perforated or perforated/stretched and solid status quo thermoplastic samples. Build‐up dose measurements were made using a parallel plate chamber. A metamaterial design was established based on a packed hexagonal geometry. Radiochromic film dosimetry was performed to determine the dependence of surface dose on the metamaterial design. Full cranial and head‐and‐neck prototype immobilizers were designed, printed, and assessed with regard to dimensional accuracy.

**Results:**

Build‐up dose measurements demonstrated the superiority of the PA12 material with a light fusing agent, which yielded a ∼15% dose reduction compared to other MJF materials. Metamaterial samples provided dose reductions ranging from 11% to 40% compared to stretched thermoplastic. MJF‐printed immobilizers were produced reliably, demonstrated the versatility of digital design, and showed dimensional accuracy with 97% of sampled points within ±2 mm.

**Conclusions:**

MJF is a promising technology for an automated fabrication of patient immobilizers. Material selection and metamaterial design can be leveraged to yield surface dose reduction of up to 40%. Immobilizer design is highly customizable, and the first examples of MJF‐printed immobilizers demonstrate excellent dimensional accuracy.

## 1 INTRODUCTION

Radiation therapy (RT) is a major treatment modality for ∼50% of all patients who have cancer. Since its inception, a range of physical patient accessories have been used for treatment delivery, including fabricated shielding blocks, treatment field‐specific and manually assembled compensators, and custom‐made molds, bolus or brachytherapy applicators. Many of these have required manual fabrication methods that may be time‐consuming in the clinic and limited with regard to control over accuracy. The field has seen a trend of these devices disappearing through digitization. For example, additive manufacturing, or 3D printing, is now used commonly to produce patient photon bolus,^1^, ^2^, ^3^, ^4^ to modulate the dose distributions of electron beams^5^ or to produce patient‐specific surface brachytherapy^6^, ^7^ or gynecological brachytherapy^8^, ^9^, ^10^, ^11^ applicators. The advantages of digitizing the design and fabrication processes include increased accuracy, quality control, and in some cases, much greater efficiency in the clinic.^12^

The status quo technology for patient immobilization is the manually fabricated thermoplastic mask. Examples of 3D printing patient immobilizers, for example, cranial or head‐and‐neck masks, are experimental^13^ and to date, no practical solution is in widespread use in clinics. However, there exist multiple motivations to provide this technology to RT facilities. Foremost may be improving upon the current patient experience with traditional thermoplastic immobilizers. Commonly and unfortunately, the mask fabrication procedure occurs near the beginning of a patient's experience in the RT clinic. Most often the thermoplastic mask material is heated using either a hot water bath or an oven, formed by hand over the skin, and the mask is left to cool and shrink on the patient's surface. Patient's anxiety associated with the mask begins early in this process: a study (*N* = 90) among head‐and‐neck patients showed that 11% of patients at CT simulation, and 24% at the time of first treatment experienced anxiety sufficient to introduce interruption of the procedure.^14^ In a separate study^15^ (*N* = 100), 20% and 6% of head‐and‐neck patients reported moderate and severe anxiety due to the mask, respectively. Regarding anxiety‐mitigating measures, almost a third of patients indicated improvements following customization of the mask, for example, creation holes around the eyes or mouth.^16^ Although masks may indeed be required during treatment delivery, the prospective digitization of the design would allow for the customization of mask based on patient attributes, including anticipated level of anxiety. Digital design would allow for the minimization of material involved, and (as we demonstrate in this work) even the customization of mask appearance, giving the patient an active role in the process. In addition, prospective per‐patient engineering design would allow for selective control over rigidity, for example, making the mask more rigid at critical points for immobilization, while relaxing the fit elsewhere to improve patient comfort. These opportunities are not afforded by today's thermoplastic immobilization.

The second motivation is the elimination of the dependence of mask quality on the manual skill of the fabricator. A poor fitting mask can result from suboptimal fabrication, and also due to changes in anatomy during the course of therapy, thus compromising patient immobilization.^17^ This uncertainty is well known in practice, and qualitative studies have indicated that a majority of health professionals cite the concern that poorly made masks may lead to poorer patient outcomes.^18^ Thus, the elimination of manual fabrication of thermoplastic masks would address a clear potential failure mode.

A third motivation relates to potential improvements of efficiency. Given the need to heat, form, and cool thermoplastic materials, the status quo approach can be time‐ and resource intensive. The fabrication may involve more than one therapist, which increases operational cost and may also consume capital resources if the fabrication is performed in the CT suite. In contrast, optical surface imaging^19^ could provide the data necessary for immobilizer design in minutes and well in advance of the CT appointment, the device could be produced in time for the CT session, without the attendance of either the patient or therapists.

For these reasons, as well as the observation of setup accuracy comparable or superior to standard devices in humans or animals,^20^, ^21^, ^22^ 3D printing of immobilizers has generated significant interest in the field, and a recent review was provided by Asfia et al.^13^ In reports where the fabrication method was disclosed, almost 60% used fused deposition modeling (FDM) printing, that is, extrusion of a heated polymer in successive layers to form an object.^13^ The frequent use of FDM likely arises from the fact that it is readily accessible and the low cost of the technology. However, FDM produces parts with limited reliability, and printing performance may depend on environmental factors such as humidity.^23^ Resolution is limited by the extruder nozzle diameter and achieving a smooth surface requires small layer heights, which increases already long print times.^23^ FDM typically requires manual postprocessing,^24^ and complex parts such as immobilizers will involve the printing of support structures that need to be removed manually. Besides FDM, other printing technologies explored for immobilization include material jetting, selective laser sintering, stereolithography, and binder jetting.^13^, [Supplementary-material acm213773-supitem-0001]

Multi jet fusion (MJF) printing has not been explored widely for this application and is distinctive from other printing technologies by several promising attributes. MJF involves spatially selective fusing of a polymer powder through the application of a fusing agent followed by the application of thermal energy. MJF is reported to be ∼10 times faster than FDM.^25^ A commonly used material printed by MJF is PA12, an engineering‐grade plastic used in a gamut of challenging applications in industry that is mechanically strong, chemically resistant to alcohols, alkalis, ethers, esters, oils, and hot water. Germane to the production of immobilizers, it is biocompatible and offers exceptional surface finish.^26^ Finally, it is among the only methods that can add a top layer in color, which may add additional advantages in radiotherapy applications.^27^

Beyond the advantages of digitizing a manual process where quality depends on human skill, if one is to develop a 3D‐printed immobilizer, this also introduces the opportunity of selecting or optimizing the materials in terms of radiological properties. Current thermoplastic materials can involve significant dose buildup due to a bolusing effect of the material on the patient surface.^28^ Among 3D printing methods, MJF is uncommonly versatile in that different fusing agents as well as various additives can be jetted that change material physical, optical, or electromagnetic properties.^29^ Thus, there may be an opportunity to select or develop low physical/electron density materials that reduce this bolusing effect. A recent publication explored the dependence of MJF color and print orientation on dose buildup and tensile strength but did not examine different possible MJF materials.^27^

The goals of this work are threefold. First, we report on the surface build‐up dosimetry for several MJF printable materials and compare directly to typical and best case alternatives for FDM printing, as well as to standard thermoplastics. Second, we propose a printed metamaterial design to be used in the fabrication of immobilizer shells with the aim of minimizing surface dose while providing mechanical rigidity and compare designs with regard to surface dose. Third, we present examples of MJF‐printed immobilizers and provide measurements of spatial accuracy for these first prototypes.

## 2 METHODS

### 2.1 MJF media candidates and comparison to common materials

**FIGURE 1:**
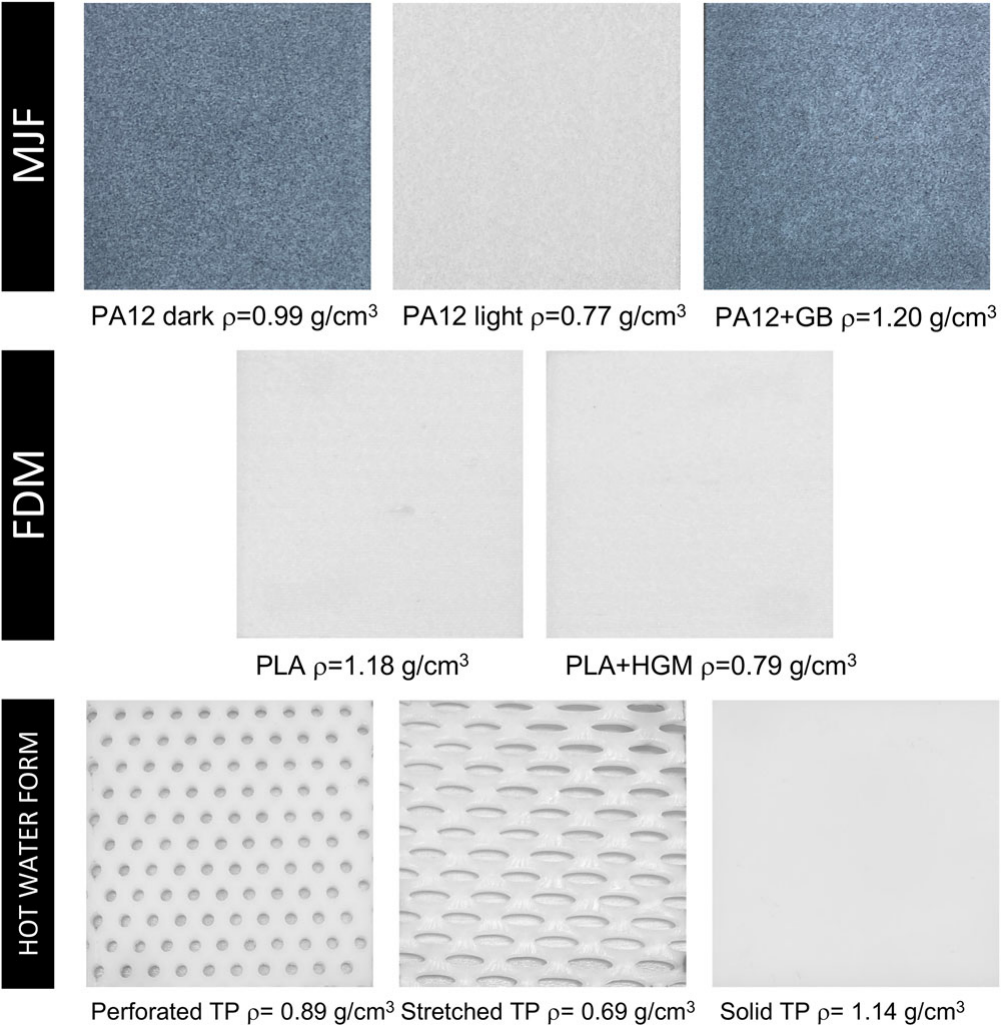
Material samples for dose build‐up measurements produced by multi jet fusion (MJF), fused deposition modeling (FDM) or by hot water‐forming thermoplastic (TP). Average measured densities of samples are indicated

In this study we considered three different media that are printable using MJF, as well as two FDM‐printable materials, with comparison to three commercially available thermoplastic immobilization samples. All printed samples were in the form of slabs ∼5 × 5 cm^2^ in area and were produced in thicknesses ranging from 1 to 3 mm. Figure 1 shows samples of each material type.

The three MJF materials were printed by the manufacturer (HP) and were all based on PA12. The first is PA12 produced with the standard fusing agent and is dark in color (commercially available as “HP 3D HR PA12” printed on the MJF 5200 printer). The second is fabricated using PA12 powder with an added component, solid glass beads, and the standard fusing agent (commercially available as “HP 3D HR PA12 GB” printed on the MJF 5200 printer). The third uses a transparent fusing agent and PA12 powder with added TiO_2_ particles. This is the standard powder used on the MJF 580 printer (“HP 3D HR CB PA12”) with a custom modification to the printing mode that is not yet commercially available in which the transparent fusing agent is applied throughout the part instead of solely on the surface. These MJF materials are henceforth labeled “PA12 dark,” “PA12+GB,” and “PA12 light,” respectively. In consultation with the manufacturer, we chose these three materials as they span a broad range of physical density (Figure 1) that allowed us to measure the dependence of surface dose on this parameter. Measured densities were 0.77, 0.99, and 1.20 g/cm^3^ for PA12 light, PA12 dark, and PA12+GB samples, respectively. Tensile, elongation, and impact mechanical properties for PA12 MJF materials have been quantified by the manufacturer^30^ and thus are not investigated in this work.

**FIGURE 2:**
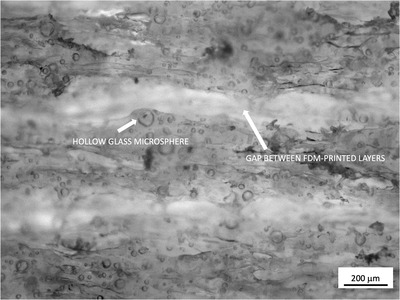
Microscopy image showing a polylactic acid+hollow glass microspheres (PLA+HGM) material in a plane perpendicular to printed layers, illustrating the presence of intact HGM, as well as gap between layers added during the fused deposition process

Although MJF is a promising technology to print immobilizers for the reasons stated earlier, FDM printing with polylactic acid (PLA) is among the most common methods used in RT clinics today for, for example, bolus fabrication or surface brachytherapy applicators. Thus, we compared the MJF candidates to PLA printed at 100% infill using a typical FDM printer (Taz 5, LulzBot). FDM slabs were printed using an extruder temperature of 220°C, bed temperature of 60°C, print speed of 15 mm/s, 0.5‐mm layer height, and 100% infill. In addition, with a view to identifying and comparing to FDM low density materials of interest for immobilization, a second FDM material was examined, produced by extruding the same PLA material in combination with hollow glass microspheres (HGM) to reduce the density of the filament. The same printer settings were used for this PLA+HGM material as for PLA alone. The HGMs were K20 glass bubbles (3 M) with a mean particle diameter of 60 μm and an average density of 0.20 g/cm^3^ according to manufacturer's specifications. Filament extrusion was done by mixing 30% HGMs by volume with pulverized PLA and extruding using an EX2 filament extruder (Filabot) into an FDM‐printable filament of diameter 2.9 mm. Microscopy imaging (Figure 2) confirmed the presence of intact HGMs following extrusion and also revealed the variability in a HGM diameter with many being smaller than the nominal 60 μm, as well as the presence of gaps between deposited layers that can be expected as a result of the FDM process (Figure 2). Measurements showed that after printing, the average density among PLA+HGM samples was 0.79 ± 0.05 g/cm^3^ compared to the density of the standard FDM slab of 1.18 g/cm^3^. We found that extrusion with HGMs above 30% by volume produced filament that did not print reliably; thus; this was the lowest density material that we could produce for FDM.

Finally, for comparison to common thermoplastics used for patient immobilization today, we included samples of solid thermoplastic, perforated thermoplastic; and stretched thermoplastic samples (CIVCO). The solid and perforated samples were 3.2 mm in thickness, whereas the stretched sample reduced the thickness to 2.4 mm and increased the perforation area by a factor of ∼3–4. This stretched thickness was based on multiple samples taken from example patient immobilizers.

### 2.2 Dose measurement for material samples

In order to measure dose below varying build‐up thicknesses of materials, we used an advanced Markus chamber (PTW Freiburg GmbH) embedded in a 10‐cm‐thick solid water phantom, with the protective cap removed. This located the reference point at the inner surface of the 0.03‐mm polyethylene entrance window, flush with the phantom surface. Measurements were made with −300‐V bias and charge collected with a Standard Imaging electrometer in integrated charge mode. Given its predominance in photon treatment of cranial and head‐and‐neck indications, a 6‐MV beam was used for all measurements, produced by a Varian TrueBeam (Varian Medical Systems) linear accelerator. A jaw‐defined 4 × 4‐cm^2^ field size was used to examine the dosimetry under narrow field conditions. This was also chosen for practical reasons, that is, to be smaller than the 5 × 5‐cm^2^ slab dimensions. For all measurements, the source‐to‐detector distance was 100 cm. Material layers were added in increments allowing high depth‐resolution (as fine as ∼1.0 mm for MJF samples) to establish ionization‐versus‐thickness curves for the 3D printed samples extending beyond a total thickness of 15 mm. As only relative measurements among samples were of interest in this work, ionization was not converted to absolute dose through calibration factors. To plot relative dose as a function of total build‐up thickness, actual slab thicknesses were measured precisely (to within 0.01 mm) using a micrometer, taking five measurements over the sample area and averaging. Given their fixed thicknesses, thermoplastic samples involved just a single layer per material type.

### 2.3 Metamaterial design for immobilizers

MJF allows exceptionally fine fabrication, with printed voxels as small as 80 μm. This capability introduces opportunities for highly detailed “metamaterial” designs that could be used for printing the shells of immobilizers, offering desirable mechanical and radiological properties. In this study, we aimed to design a metamaterial for immobilization yielding mechanical rigidity while minimizing the amount of material required. A key requirement for our design was an extremely thin shell to reduce skin dose. A likely design candidate for the metamaterial is a hexagonal/honeycomb structure printed on a solid face or sandwiched between two faces. This choice arises from nature: Since ∼36 BC, it has been speculated that bees’ hexagonal structure minimizes material in filling the honeycomb while providing a highly rigid structure. Moreover, a mere 20 years ago, *The Honeycomb Conjecture* ^31^ provided the mathematical proof that this structure minimizes total perimeter (and in our case, material) in partitioning a plane into shapes of equal area. Our selection of a hexagonal structure for MJF‐printed metamaterial also draws upon numerous examples of creating light and stiff structures in aeronautical, automotive and marine applications, among others.

**FIGURE 3:**
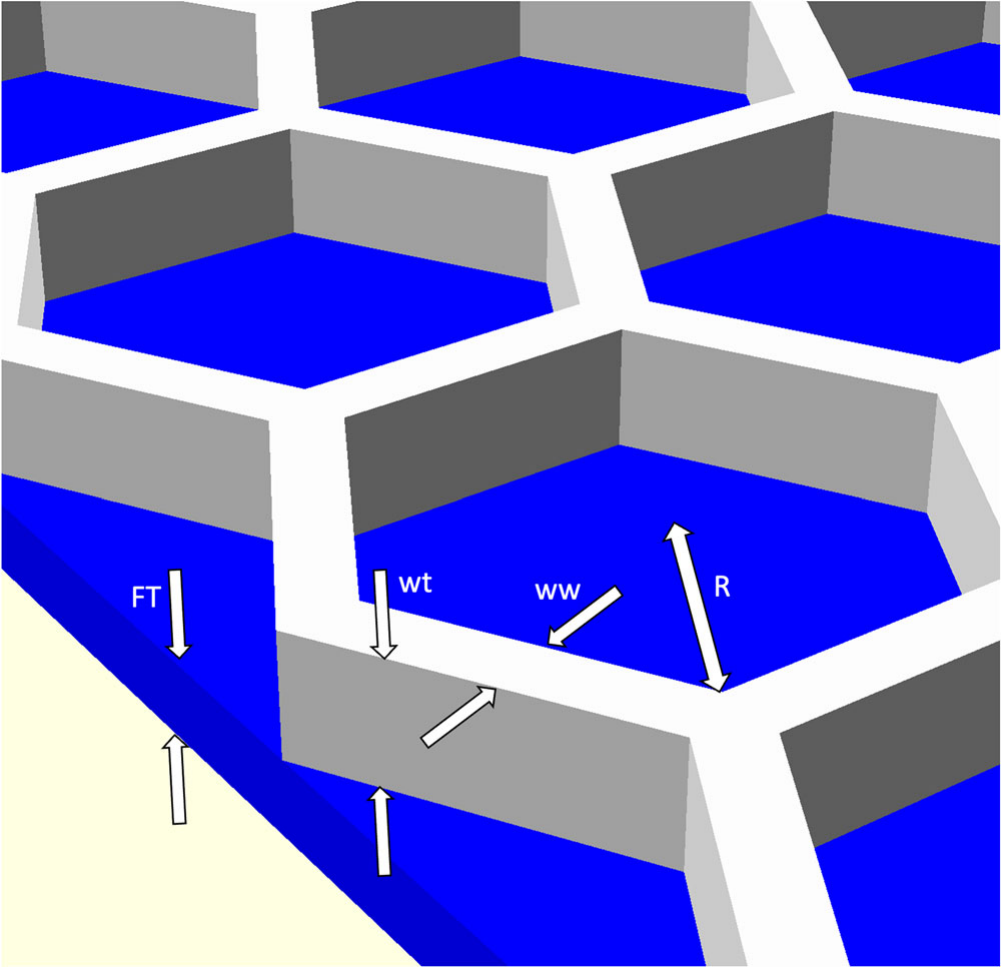
Parameterization of the hexagonal metamaterial, where *FT* is the face thickness, *ww* is the wall width, *wt* is the wall thickness, and *R* is half of the inner long diagonal

In exploring the dosimetric consequences of various hexagonal geometries, we define the parameters as shown in Figure 3. *FT* is the face thickness, that is, the thickness of the skin of material underlying or, in the case of a dual‐face structure, also overlying the walls of hexagons. The width of hexagonal walls is *ww* and the wall thickness is *wt*. The total thickness is *T*, equal to the sum of *FT* and *wt*. *R* is half the length of the inner long diagonal.

Of course, limitless combinations of these parameters are possible. We selected representative values that would, at one extreme, minimize the total amount of material used and, at the other, would produce a highly mechanically robust design. For example, *FT* was set to either 0.4 mm to provide an extremely thin face thickness and to minimize skin dose while providing some mechanical rigidity, or alternately to 1.0 mm, which approaches the thickness of highly stretched conventional thermoplastic. The cell radius *R* was chosen to be 6 mm as this is small enough that it can wrap around facial features without causing design complications and is comparable to some of the perforation sizes seen in conventional thermoplastic immobilizers.

**FIGURE 4:**
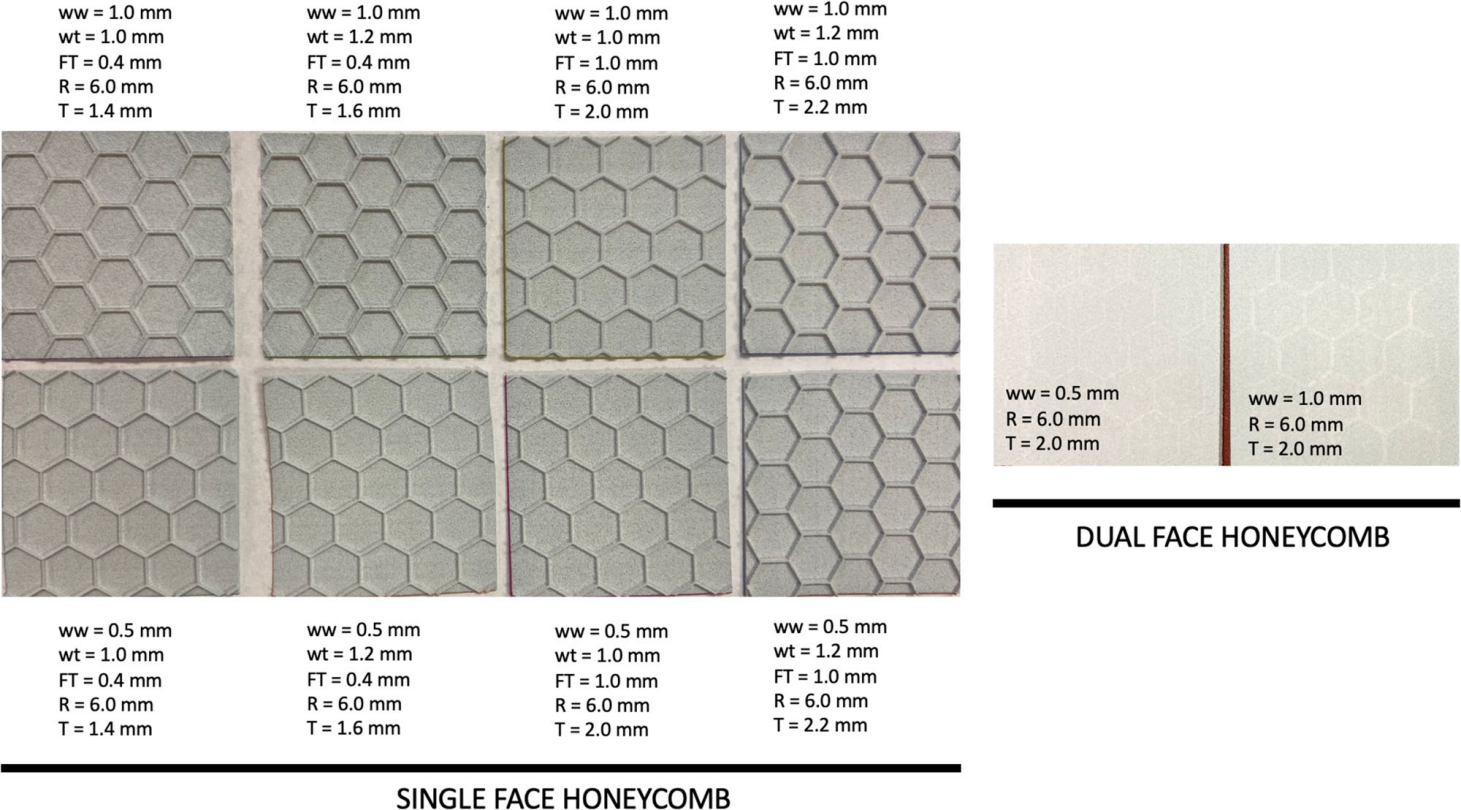
The 10 metamaterial designs examined in this work, varying values of face thickness (*FT*), wall thickness (*wt*), wall width (*ww*). Cell radius (*R*) was fixed at 6.0 mm

Figure 4 shows images of all 10 metamaterial samples produced, where each sample was 5 × 5 cm^2^ in area. The majority of these designs included a single‐face rather than a dual‐face design, that is, one with skins sandwiching the hexagonal structure. Although a dual‐face design would likely provide more rigidity, we anticipated that this approach might trap MJF polymer powder between faces during fabrication, thus increasing the overall radiological path length, and compromising low‐dose build‐up characteristics. In addition, from a patient‐focused/clinical point of view, we foresee a future need to design perforated metamaterials, which may be incompatible with the dual‐face design.

### 2.4 Dose measurement for metamaterial samples

Given the complex geometry of the metamaterial, one can anticipate spatial variability in the surface dose measured, that is, a higher build‐up dose below the hexagonal walls with total thickness *T* relative to regions below smaller thickness *FT*. To measure this variation and to quantify the average build‐up dose between samples, we employed a radiochromic film dosimeter type EBT3 (Ashland), which provides a dynamic range of 0.2–10 Gy. For each exposure, a metamaterial slab was placed directly on top of the radiochromic medium, which in turn was positioned on the surface of a 10 cm‐thick solid water phantom. Each exposure was conducted with 200 MU and a 6 × 6‐cm^2^ field. Films were digitized using an Epson 10000XL transparency scanner^32^ maintaining consistency in film orientation on the scanner bed. Calibration was performed according to the manufacturer, that is, exposing a range of film strips to known doses. All three RGB channels were used for calibration, and self‐consistency between these channels was verified. The calibration comprised a dose range between 20 and 300 cGy. It was verified that measured dose values under both the *FT* and *T* thicknesses, for all samples, were in the calibrated range. In all cases, calibration and films for metamaterial samples were taken from the same film batch and were scanned and analyzed in the same session, waiting 24 h between exposure and readout.

### 2.5 Design and fabrication of cranial and head‐and‐neck immobilizers

To design realistic prototype cranial and head‐and‐neck immobilizers, we CT imaged the male atom anthropomorphic phantom (CIRS) positioned on an S‐frame immobilizer baseplate (CIVCO) and Silverman B head rest. The CT slice thickness was 2.5 mm. Based on these image data, the surface was defined using thresholding (HU > −800), and solid model design software (Blender) was used to create immobilizer geometries. An algorithmic software workflow was used to re‐mesh the surface data, tile the hexagonal metamaterial to conform to the phantom geometry, add mouth and eye holes, and integrate features that connect to the S‐frame immobilizer base plate. Many of these processes involved manual manipulation during development but can be automated in software as the design stabilizes. Multiple versions of immobilizers were created, including two different metamaterials with *FT* of 0.4 and 1.0 mm, *wt* of 1.0 mm, and *ww* of 1.0 mm. In addition, both cranial and head‐and‐neck versions were designed, for demonstration purposes.

To assess the accuracy of the MJF‐printed immobilizers, we acquired three‐dimensional surface data using HP 3D Scan (HP). This imaging system includes a projector that casts structured light on the scanned object, two cameras for acquisition, and a rotation stage to capture views of the imaged object through 360°. The system provides 0.05‐mm spatial sampling with up to 2.3 × 10^6^ vertices per scan. The system software allows direct comparison between the design file and the scanned surfaces, providing a frequency histogram of three‐dimensional spatial deviation.

## 3 RESULTS

### 3.1 Dose measurement for material samples

**FIGURE 5:**
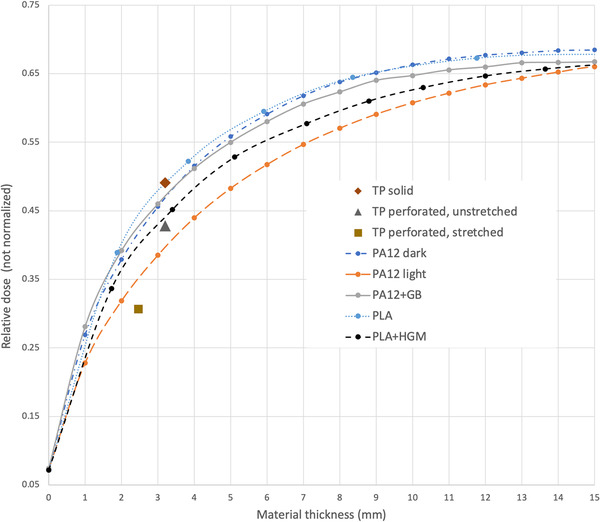
Relative dose measured as a function of material thickness for multi jet fusion (MJF) PA12 samples, FDM polylactic acid (PLA) samples, and single layers of solid, perforated, and perforated/stretched thermoplastic (TP)

**FIGURE 6:**
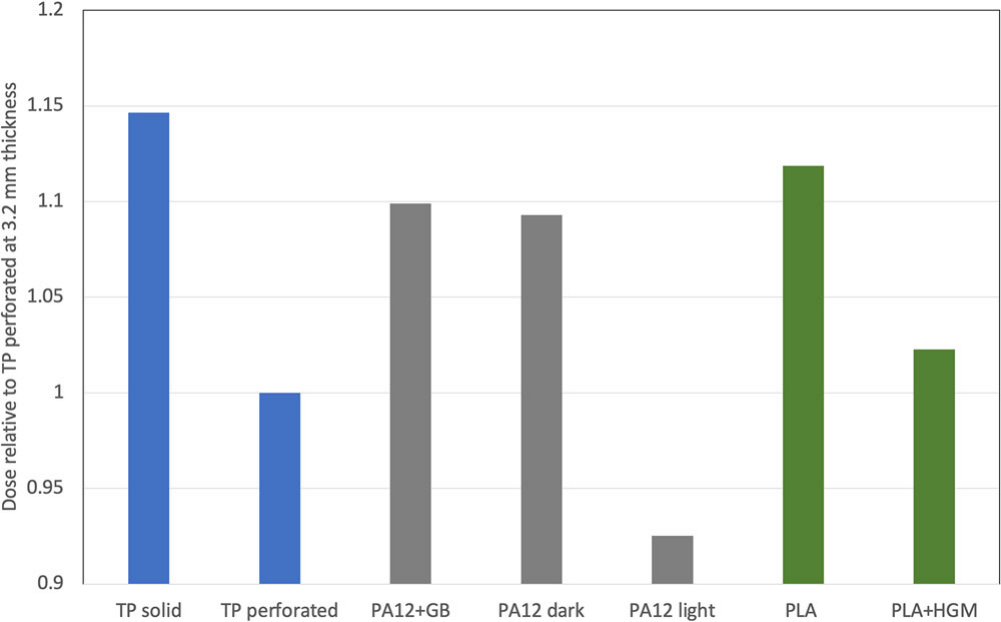
Dose relative to perforated (unstretched) thermoplastic, for samples at a thicknesses of 3.2 mm, that is, the thickness of stock thermoplastic materials

Figure 5 shows measured relative dose as a function of added thickness of various sample materials, where measurements were not normalized, allowing a comparison of absolute values. At thickness values comparable to conventional masks, for example, 3 mm or less, PA12 dark, PA12+GB and standard FDM‐printed PLA involve dose values within 5% of each other. The presence of HGM in PLA causes a significant reduction of dose, whereas the MJF‐printed PA12 light provides the lowest value among all solid samples tested. Conventional solid thermoplastic yields dose comparable to that for PLA at the same thickness. Interestingly, although perforated, thermoplastic dose remains higher than that for a solid sample of PA12 light of the same thickness. Stretching the thermoplastic from 3.2 to 2.4 mm produces a sample with ∼12% lower dose than a solid sample of PA12 light at the same thickness. The PA12 light material is also superior to the low‐density FDM‐printed sample containing HGMs. Figure 6 provides a summary of measured relative dose values at a common thickness of 3.2 mm, corresponding to the stock thickness of perforated and solid thermoplastics. PA12 light appears to be a promising material for application to immobilizers, that is, even without perforations it provides a lower surface dose.

### 3.2 Dose measurement for metamaterial samples

**FIGURE 7:**
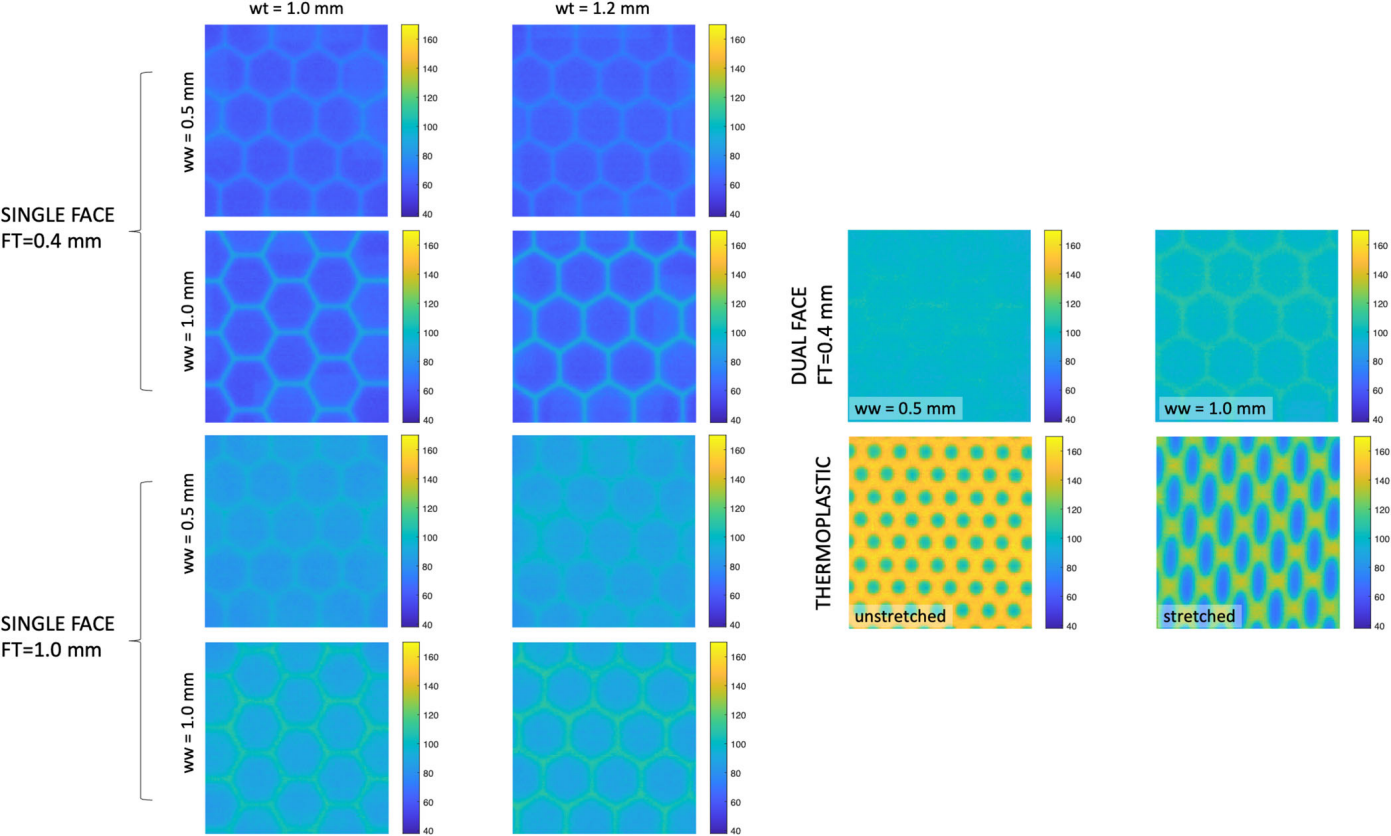
Shown on left, images from radiochromic dosimetry under single‐face metamaterial samples. Shown on right, dose images for dual‐face metamaterial samples as well as unstretched and stretched perforated thermoplastic. Color bars show calibrated dose in cGy

Figure 7 shows images resulting from radiochromic dosimetry of the metamaterial samples after calibration to dose. Shown on the left are the various samples with a single‐face design, for various combinations of *FT*, *wt*, and *ww*. All images are shown with the same dose color scale for comparison. This demonstrates the effect of various metamaterial design parameters on dose, with single‐face geometries providing much lower dose compared to dual‐face designs shown on the right. Upon investigation, it was confirmed that the dual‐face designs indeed trapped PA12 powder between face layers, which increases dose buildup significantly. All examples of metamaterial samples provide lower dose than either unstretched or stretched thermoplastic samples.

**FIGURE 8:**
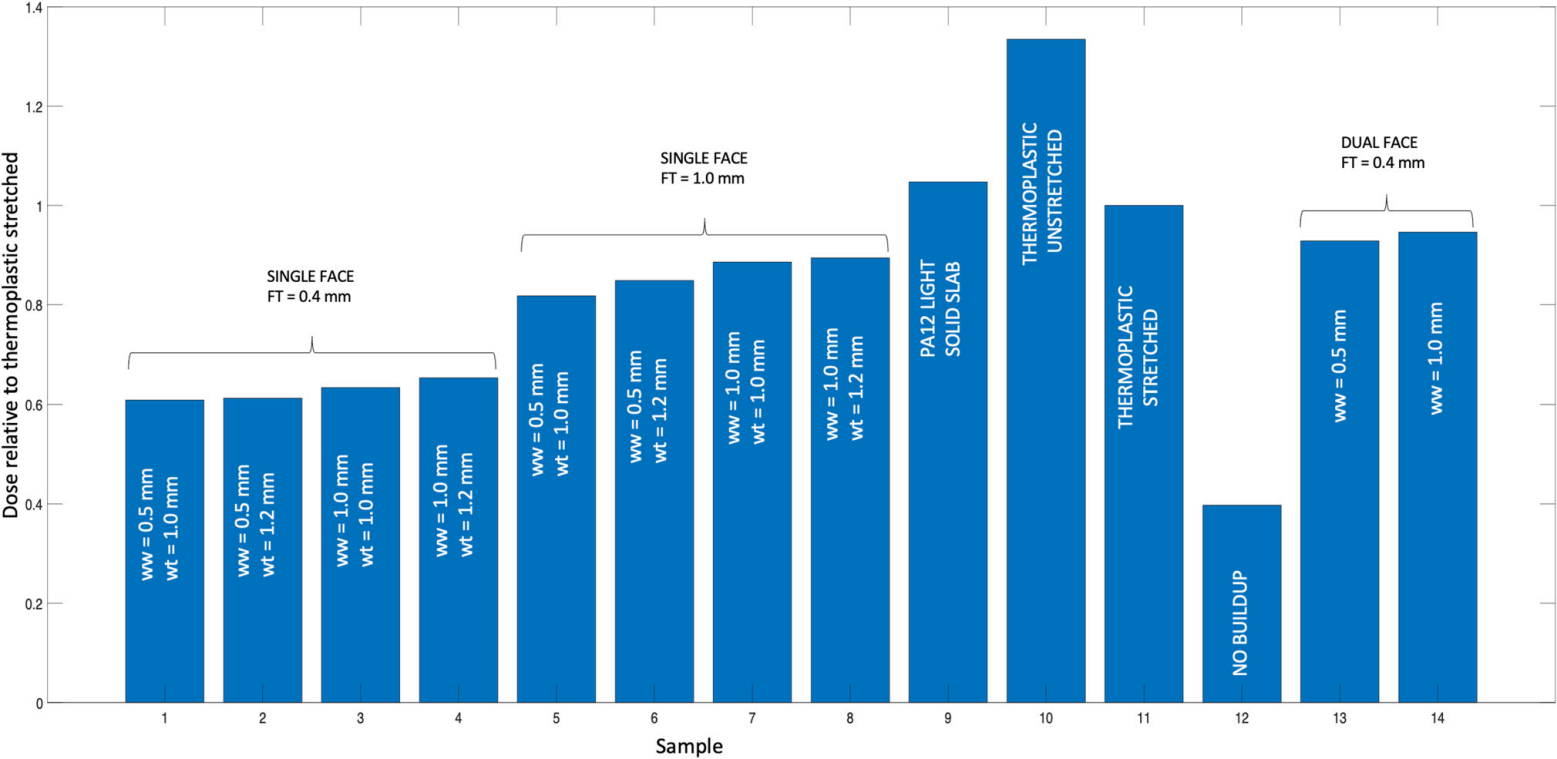
Measured dose relative to perforated/stretched thermoplastic for all metamaterial samples

Calculating average dose over the area of each sample provides the results shown in Figure 8. Here, for practical comparison, all dose values have been normalized to that of the status quo, that is, stretched thermoplastic. In summary, dual‐face metamaterial designs provide a dose reduction by as much as 7% compared to stretched thermoplastic. Single‐face designs are far superior, providing significant dose reductions: by 11% to 18% for *FT* of 1.0, and by 35% to 40% for *FT* of 0.4 mm. The effect of varying *wt* and *ww* is modest, increasing these parameters produces increase of dose by 5% to 7% for a fixed value of *FT*.

### 3.3 Design and fabrication of cranial and head‐and‐neck immobilizers

**FIGURE 9:**
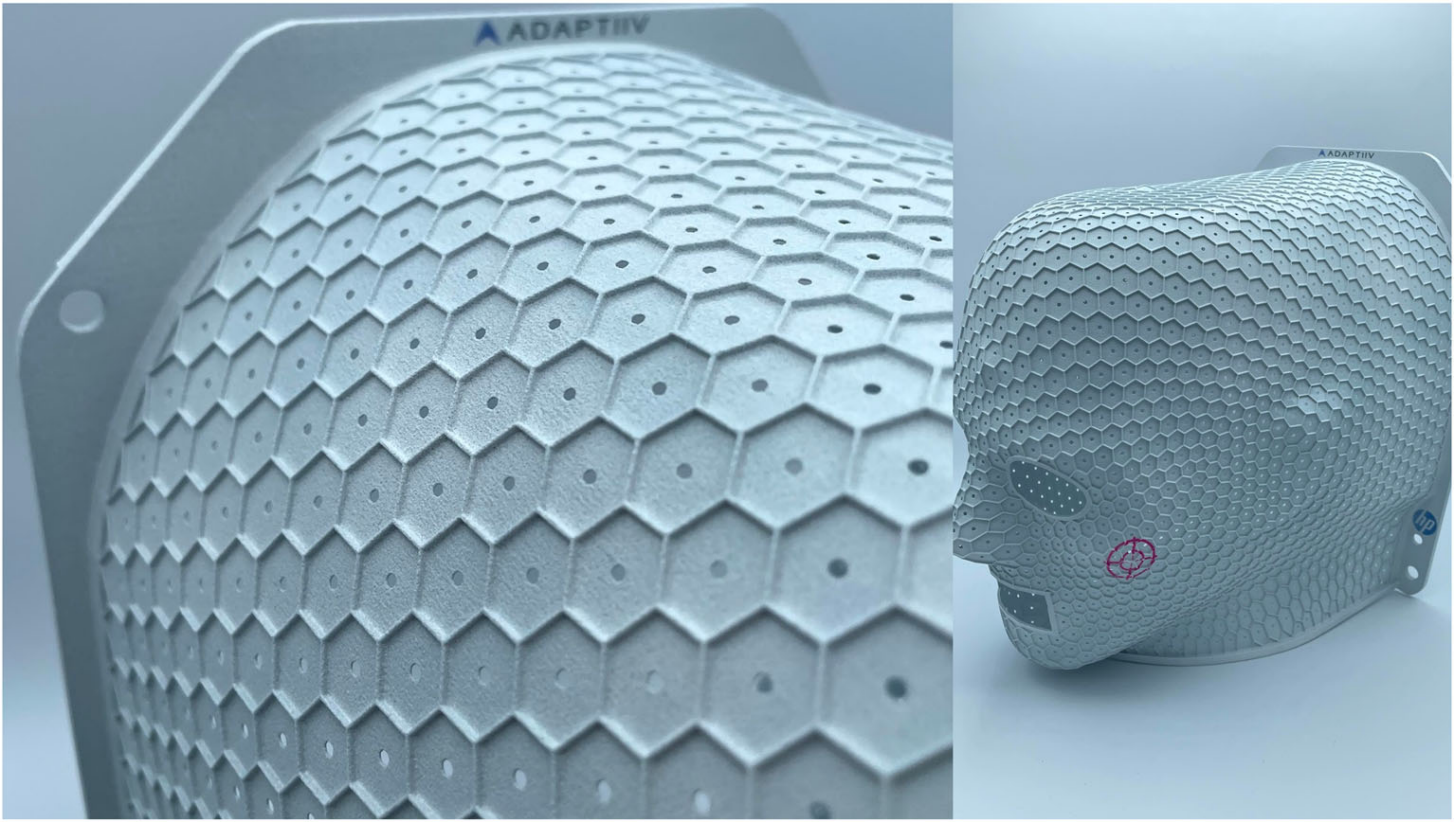
On the left, a multi jet fusion (MJF)‐printed immobilizer showing close‐up of the metamaterial design with added perforations. Shown on the right, the design includes a crosshair as an example of the utility of color printing in this application. This immobilizer was produced with *R* of 6 mm, although this value is variable with stretching around surface curvature. *FT*, *ww*, and *wt* were 0.4, 1.0, and 1.0 mm, respectively. Perforations had a radius of 1 mm

**FIGURE 10:**
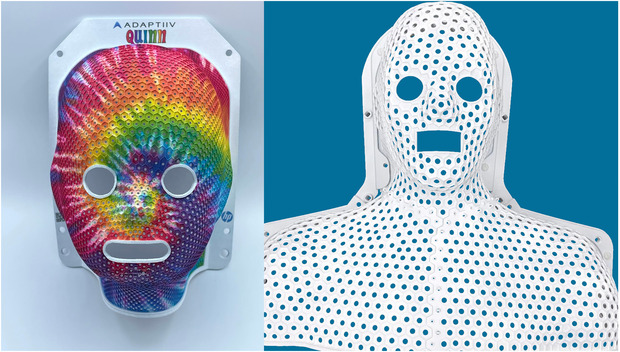
On the left, an example of a pediatric cranial immobilizer using the metamaterial design and printed pattern on the surface. On the right, a full head‐and‐neck immobilizer incorporated into a frame that is compatible with an S‐type immobilizer base plate. The pediatric immobilizer was produced with an *R* of 3 mm, although this value is variable with stretching around surface curvature. *FT, ww*, and *wt* were 0.4, 0.5, and 0.6 mm, respectively, and perforations were 2 mm in radius. The head‐and‐neck immobilizer was designed with an *R* of 6.0 mm, with *FT, ww*, and *wt* of 1.0 mm, with 3.0‐mm radius perforations

Figure 9 shows an example of a cranial immobilizer produced using MJF printing with a PA12 light material. Here, the prototype incorporates the metamaterial design as described earlier and illustrates the option of adding perforations centered on each hexagonal cell. These perforations would be desirable, for example, to improve air flow, to allow the therapist to assess distance from the shell to the skin to detect anatomical change, and to provide a device that is potentially less claustrophobic for the patient. The perforations may be customizable during digital design. Figure 10 shows additional examples, including a pediatric cranial immobilizer, where the option of color printing has been used in a custom design. Also shown is an extended head‐and‐neck immobilizer. In this example, the patient‐specific immobilizer has been integrated into a 3D‐printed frame that is compatible with the anchor points of an S‐frame type base plate.

**FIGURE 11:**
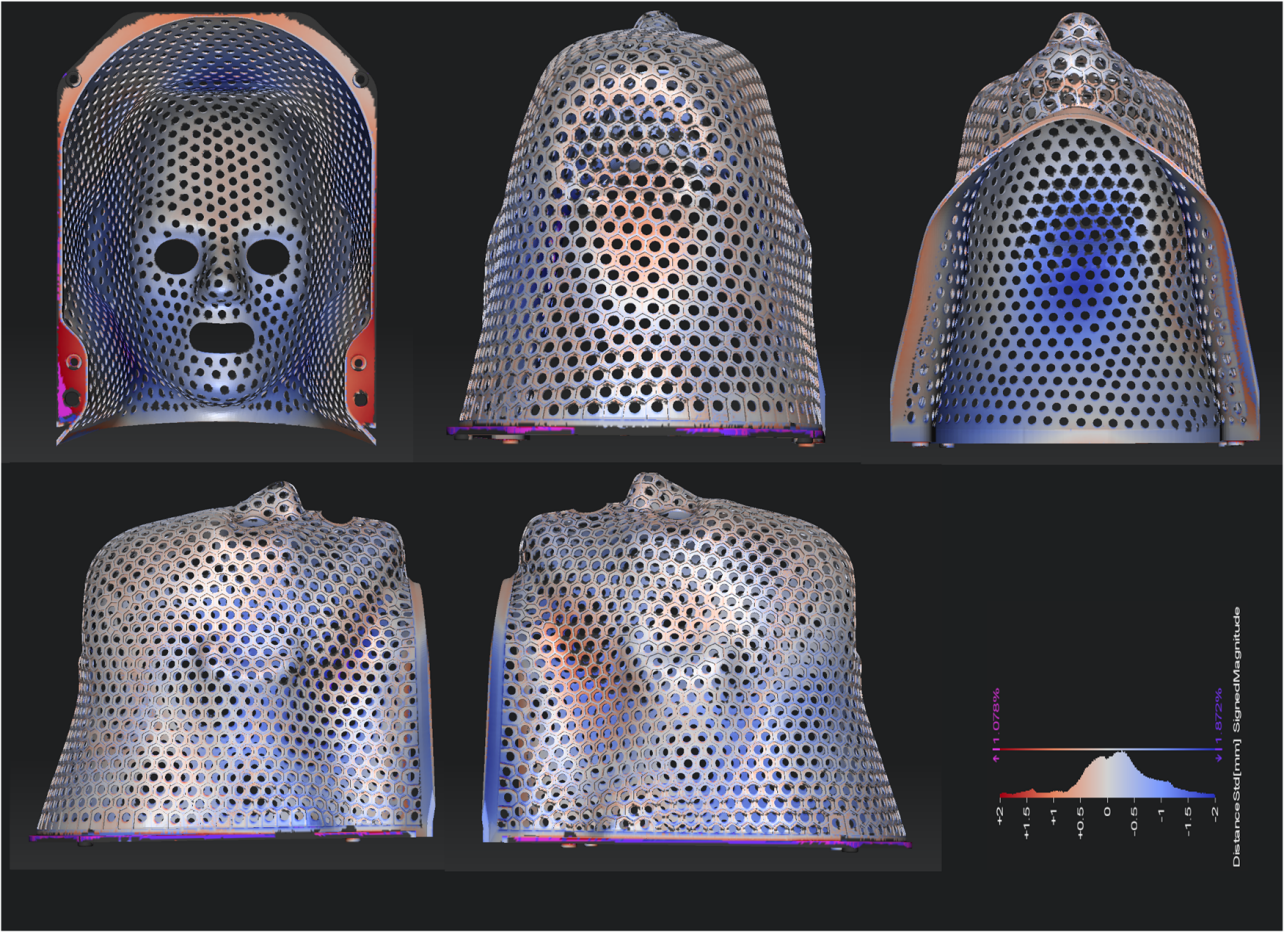
Optical scan data of adult cranial immobilizer, where color scale shows spatial deviation from design dimensions. Color scale ranges between ±2 mm between red and blue, with white corresponding to zero deviation. This immobilizer was designed with an *R* of 6.0 mm, with *FT*, *ww*, and *wt* of 1.0 mm, and with 3.0‐mm radius perforations

**FIGURE 12:**
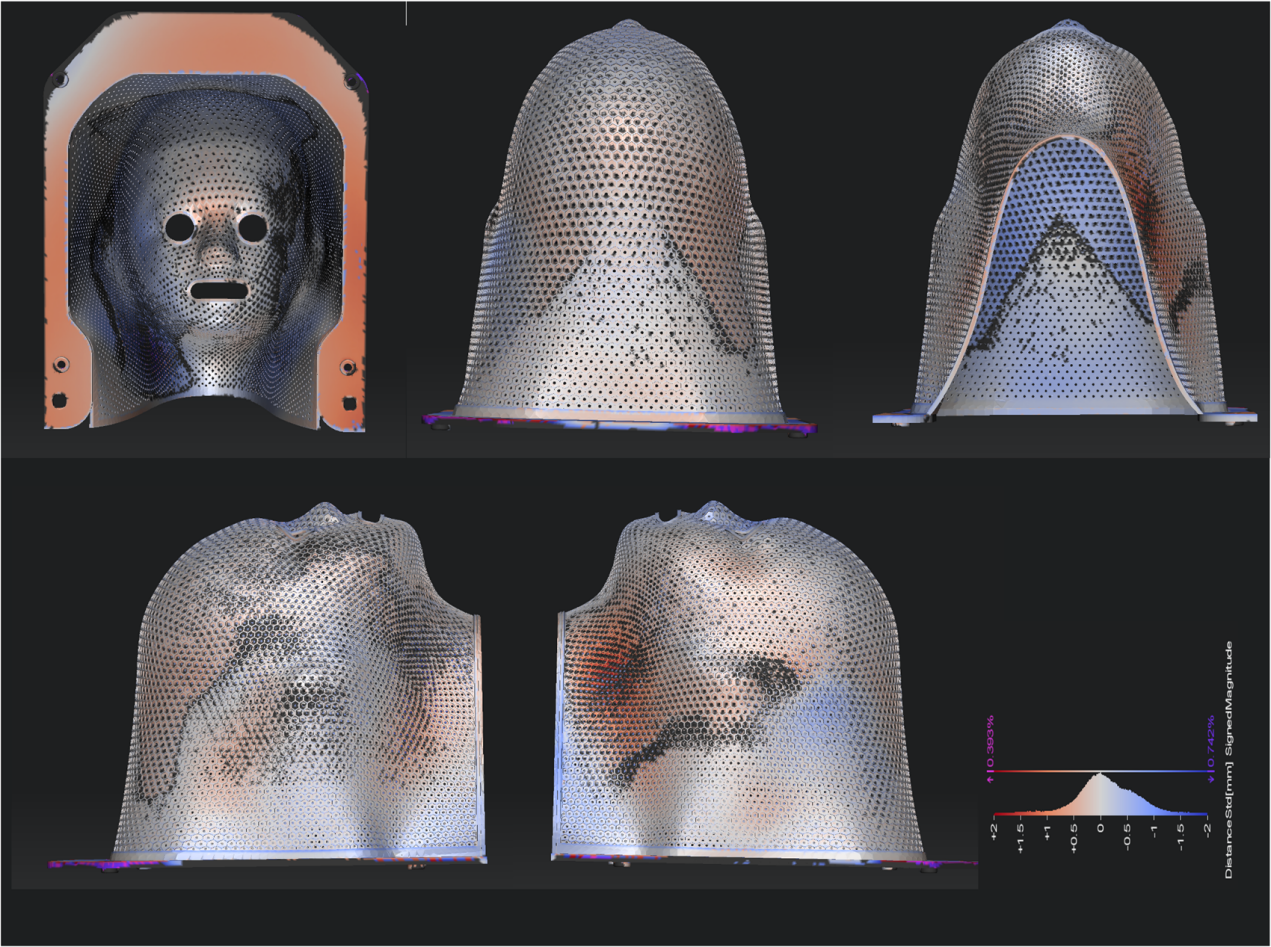
Optical scan data of a pediatric cranial immobilizer, where the color scale shows spatial deviation from design dimensions. Color scale ranges between ± 2 mm between red and blue, with white corresponding to zero deviation. This pediatric immobilizer was produced with an *R* of 3 mm. *FT*, *ww*, and *wt* were 0.4, 0.5, and 0.6 mm, respectively. Perforations were 0.75 mm in radius

Figures 11 and 12 provide the results of assessment of the dimensional accuracy of printed adult and pediatric immobilizers, that is, comparing the optical scan surface to that in the design file. Here the color scale depicts deviation between ±2 mm between red and blue, with white indicating zero deviation. For the adult immobilizer, 83.4%, 97.1%, and 98.9% of sampled points show dimensional accuracy within ±1, ±2, and ±3 mm, respectively. For the pediatric immobilizer, the corresponding values are 92.5%, 98.9%, and 99.7%. We note that these values are based on a best fit between the designed and optically scanned surfaces and exclude the frame, which is a generic component.

## 4 DISCUSSION

This study has explored the potential of MJF 3D printing for the fabrication of patient immobilizers, with a focus on the impact of various MJF materials and metamaterial design on dose buildup. Aside from the benefits of digitizing/automating the design and fabrication, a material and design that lowers surface dose would be advantageous for multiple treated sites. In head‐and‐neck radiotherapy, grade 1–4 skin toxicity is experienced by patients, and although the causes may be multifactorial,^33^ the unwanted bolus effect of the immobilizer is consistently cited as a cause of increased skin dose.^28^, ^33^, ^34^ In addition, immobilization is used in treatment of multiple extracranial sites, including intact breast, where erythema and desquamation are common toxicities and conventional thermoplastic materials may increase skin dose by over 50%.^35^

Our results identify the PA12 light material as preferable with regard to dosimetry as it will provide a ∼15% surface dose reduction compared to the other solid MJF materials examined. In fact, the measured dose for this material was lower than that resulting from conventional perforated thermoplastic of the same thickness. When combined with the hexagonal metamaterial design shown here, relative to stretched thermoplastic used currently in the clinic, dose reductions ranging from 11% to 40% should be realized, largely depending on the face thickness of the hexagonal structure. For these dosimetric measurements, we did not perforate metamaterial samples, as it may be desired to print some regions of immobilizers without perforation, and we were interested in “worst case” comparisons relative to standard perforated thermoplastic. It can be expected, however, that perforation would lower the average build‐up dose further.

A study by Asfia^27^ examined the possible influence of printed color and printing orientation on surface dose but did not examine different MJF materials. At 6 MV, a common photon energy for both cranial and head‐and‐neck treatments, no dependence of dosimetry was found on five possible colors. Tensile strength was found to depend on print orientation, for example, by ∼15% for a 2‐mm sample, with the 45° print orientation preferable.

Our study did not measure mechanical properties of the PA12 samples as this has been reported on in‐depth with comparison to other 3D printing methods^36^ and already quantified in terms of tensile, elongation, and impact properties.^30^ MJF‐printed PA12 has a tensile strength of 50 MPa^30^ that is higher than that of many plastics, including ABS, polyethylene, polypropylene, and polystyrene. We observed the immobilizers produced in this work to be robust and resilient to repeated applications to an anthropomorphic phantom. As immobilizer designs are refined, it will be necessary to assess the mechanical robustness of devices as a whole, for example, to identify potential failure modes that may be due to the design rather than the inherent mechanical properties of the metamaterial. In addition, although the dimensional accuracy of immobilizers was found to be excellent with over 97% of sampled points within ±2 mm, assessing the degree of fit to human subjects will be important, as well as the ultimate performance of the immobilizers with respect to positioning accuracy. These topics will be subjects of future studies by our collaboration.

Aside from questions on immobilizer material, design, and fabrication, a number of process issues will need to be addressed to provide viable solutions for the clinic. If CT data are to be used as the input to immobilizer design, the fit may be dependent on the HU threshold used for surface definition, and further investigation would be required to refine this value. However, in general, the use of CT may be limiting from a workflow perspective. For example, 3D surface data will be required to design the device, yet a final treatment planning CT will be required with the patient positioned in the immobilizer. The option of repeating CT imaging would be suboptimal due to cost and inconvenience. However, optical surface imaging is fast, does not involve ionizing radiation, and provides excellent spatial fidelity.^19^, ^37^, ^38^ One can envisage acquiring these data at the time of first consultation in the clinic with the patient positioned on the immobilization baseplate and headrest, for example. Next, a practical software design application will be needed, to offer a programmatic, efficient, and robust workflow, that is, the surface data must drive the design without the need for extensive design skills by the user. At this point, customization (eye, mouth holes, color patters, and labeling) would be applicable. Finally, regarding timing of production and receipt by the facility, the immobilizers in this study were produced within 1 day. Thus, this may allow for a 2‐day turnaround between design/order of the device and receipt, allowing the immobilizer to be available in time for a CT simulation appointment. With these provisions, the various advantages of 3D printed immobilization, including consistent quality, accuracy of fit, flexibility of design, and automation of processes, may be realized in the clinic.

## 5 CONCLUSION

This is the first investigation to examine various MJF‐printed materials from the point of view of the surface dosimetry of patient immobilizers. Three PA12 materials were considered, spanning a wide range of density, from 0.77 to 1.20 g/cm^3^. Build‐up dose measurements demonstrated the superiority of the PA12 material with a light fusing agent, which, at the thickness of unstretched thermoplastic, produced a lower dose even with a non‐perforated sample. This material was also superior to FDM‐printed samples, even after lowering the density of PLA using HGM. We introduced a novel metamaterial design based on a hexagonal geometry and showed that relative to typical stretched thermoplastic samples, dose reductions of up to 40% may be achieved. For the first time, novel MJF‐printed immobilizers have been demonstrated, with adult and pediatric cranial examples, as well as extended masks for head‐and‐neck immobilization. The MJF‐printed immobilizers demonstrated excellent dimensional accuracy, with over 97% of sampled points within ±2 mm.

## AUTHOR CONTRIBUTION

James L. Robar: conception of technology, experimental design, measurements, analysis, manuscript writing

Barret Kammerzell, Kevin Hulick, Pierre Kaiser, Calvin Young, Vanessa Verzwyvelt, Xin Cheng, Matthew Shepherd, James Stasiak: MJF material science, immobilizer development

Radojka Orbovic, Sara Fedullo: conducted dosimetric measurements

Stephen DiMarco: software design of immobilizers

## CONFLICT OF INTEREST

The lead author (JR), a professor of radiation oncology, is also a cofounder of Adaptiiv Medical Technologies, a company developing 3D‐printed technologies in radiation oncology, and thus holds financial interest. Four coauthors are scientists and/or engineers at Adaptiiv Medical Technologies. The remaining authors are scientists and/or engineers at divisions of HP and HP Labs that specialize in MJF 3D printing.

## Supporting information

Supporting Information
