# Supplementary material for: Novel multi jet fusion 3D‐printed patient immobilization for radiation therapy
Source: J Appl Clin Med Phys. 2022 Sep 2;23(11):e13773. doi: 10.1002/acm2.13773 (PMC9680581; doi:10.1002/acm2.13773)
Supplement: Supplementary file 1 — Supporting Information [file ACM2-23-e13773-s001.pdf]

Novel Multi Jet Fusion 3D printed patient immobilization for radiation therapy

James L. Robar[1,2,5]
Barret Kammerzell[4]
Kevin Hulick[4]
Pierre Kaiser[4]
Calvin Young[4]
Vanessa Verzwyvelt[4]
Xin Cheng[4]
Matthew Shepherd[4]
Radojka Orbovic[5]
Sara Fedullo[5]
Christopher Majcher[5]
Stephen DiMarco[5]
James Stasiak[3]

1 Department of Radiation Oncology, Dalhousie University, Halifax, Canada
2 Nova Scotia Health, Halifax, Canada
3 HP Labs, Corvallis OR, USA
4 HP, Vancouver WA and Corvallis OR, USA
5 Adaptiiv Medical Technologies, Halifax, Canada

Corresponding author:
James L. Robar, PhD, FCCPM
Department of Radiation Oncology
Dalhousie University
5820 University Avenue
Halifax, Canada

Suggested running title:
3D printed immobilization for RT

Author contribution statement:
JLR: conception of technology, experimental design, measurements, analysis, manuscript writing
BK, KH, PK, CY, VV, XC, MS, JS: MJF material science, immobilizer development
RO, SF: Conducted dosimetric measurements
SD: Software design of immobilizers

(Removed from manuscript for double-blinding)

Acknowledgements

The authors gratefully acknowledge Ms. Angela Henry for proofreading and editing of the manuscript.

Conflict of interest

The lead author (JR), a professor of radiation oncology, is also a co-founder of Adaptiiv Medical Technologies, a company developing 3D printed technologies in radiation oncology, and thus holds financial interest. Four co-authors are scientists and/or engineers at Adaptiiv Medical Technologies. Remaining authors are scientists and/or engineers at divisions of HP and HP Labs that specialize in MJF 3D printing.

Data availability statement

The data that support the findings of this study are available from the corresponding author upon reasonable request.
